# Comparison of Patients Hospitalized With Influenza A Subtypes H7N9, H5N1, and 2009 Pandemic H1N1

**DOI:** 10.1093/cid/ciu053

**Published:** 2014-01-31

**Authors:** Chen Wang, Hongjie Yu, Peter W. Horby, Bin Cao, Peng Wu, Shigui Yang, Hainv Gao, Hui Li, Tim K. Tsang, Qiaohong Liao, Zhancheng Gao, Dennis K. M. Ip, Hongyu Jia, Hui Jiang, Bo Liu, Michael Y. Ni, Xiahong Dai, Fengfeng Liu, Nguyen Van Kinh, Nguyen Thanh Liem, Tran Tinh Hien, Yu Li, Juan Yang, Joseph T. Wu, Yaming Zheng, Gabriel M. Leung, Jeremy J. Farrar, Benjamin J. Cowling, Timothy M. Uyeki, Lanjuan Li

**Affiliations:** 1 Institute of Respiratory Medicine, Beijing Hospital, National Health and Family Planning Commission; 2 Department of Respiratory Medicine, Capital Medical University; 3 Beijing Institute of Respiratory Medicine; 4 Beijing Key Laboratory of Respiratory and Pulmonary Circulation Disorders; 5 Division of Infectious Disease, Key Laboratory of Surveillance and Early Warning on Infectious Disease, Chinese Center for Disease Control and Prevention, Beijing, China; 6 Oxford University Clinical Research Unit–Wellcome Trust Major Overseas Programme, Hanoi, Vietnam; 7 Centre for Tropical Medicine, Nuffield Department of Clinical Medicine, Oxford University, Oxford, United Kingdom; 8 Singapore Infectious Disease Initiative; 9 Beijing Chao-Yang Hospital, Beijing Institute of Respiratory Medicine, Capital Medical University, Beijing; 10 Division of Epidemiology and Biostatistics, School of Public Health, Li Ka Shing Faculty of Medicine, University of Hong Kong, Hong Kong Special Administrative Region; 11 Collaborative Innovation Center for Diagnosis and Treatment of Infectious Diseases, Hangzhou; 12 State Key Laboratory for Diagnosis and Treatment of Infectious Diseases, Department of Infectious Diseases, the First Affiliated Hospital, College of Medicine, Zhejiang University, Hangzhou; 13 Department of Respiratory and Critical Care Medicine, Peking University People's Hospital, Beijing, China; 14 National Hospital for Tropical Diseases; 15 National Hospital for Pediatrics, Hanoi; 16 Hospital for Tropical Diseases, Ho Chi Minh City, Vietnam; 17 ISARIC, Centre for Tropical Medicine, University of Oxford, Churchill Hospital, Oxford, United Kingdom; 18 Influenza Division, National Center for Immunization and Respiratory Diseases, Centers for Disease Control and Prevention, Atlanta, Georgia

**Keywords:** influenza A(H7N9), influenza A(H5N1), clinical epidemiology

## Abstract

***Background.*** Influenza A(H7N9) viruses isolated from humans show features suggesting partial adaptation to mammals. To provide insights into the pathogenesis of H7N9 virus infection, we compared risk factors, clinical presentation, and progression of patients hospitalized with H7N9, H5N1, and 2009 pandemic H1N1 (pH1N1) virus infections.

***Methods.*** We compared individual-level data from patients hospitalized with infection by H7N9 (n = 123), H5N1 (n = 119; 43 China, 76 Vietnam), and pH1N1 (n = 3486) viruses. We assessed risk factors for hospitalization after adjustment for age- and sex-specific prevalence of risk factors in the general Chinese population.

***Results.*** The median age of patients with H7N9 virus infection was older than other patient groups (63 years; *P* < .001) and a higher proportion was male (71%; *P* < .02). After adjustment for age and sex, chronic heart disease was associated with an increased risk of hospitalization with H7N9 (relative risk, 9.68; 95% confidence interval, 5.24–17.9). H7N9 patients had similar patterns of leukopenia, thrombocytopenia, and elevated alanine aminotransferase, creatinine kinase, C-reactive protein, and lactate dehydrogenase to those seen in H5N1 patients, which were all significantly different from pH1N1 patients (*P* < .005). H7N9 patients had a longer duration of hospitalization than either H5N1 or pH1N1 patients (*P* < .001), and the median time from onset to death was 18 days for H7N9 (*P* = .002) vs 11 days for H5N1 and 15 days for pH1N1 (*P* = .154).

***Conclusions.*** The identification of known risk factors for severe seasonal influenza and the more protracted clinical course compared with that of H5N1 suggests that host factors are an important contributor to H7N9 severity.

## Abstract (precis)

Hospitalization with H7N9 virus infection is associated with older age and chronic heart disease, and patients have a longer duration of hospitalization than patients with H5N1 or pH1N1. This suggests that host factors are an important contributor to H7N9 severity.

**(See the Editorial Commentary by Hui and Hayden on pages 1104–6.)**

The emergence of human infections with avian influenza A(H7N9) virus further widens the spectrum of novel influenza A viruses that currently pose a threat to public health [1]. Although H7N9 virus has not been shown to transmit efficiently between humans, there are indications that the recently emerged H7N9 viruses are better adapted to replication in mammalian cells than other avian influenza A viruses and represent a plausible pandemic threat [2, 3]. H7N9 viruses isolated from human cases have amino acid sequences in the hemagglutinin (HA) protein that are associated with improved binding to α2–6-linked sialidases that are abundant on human respiratory epithelial cells, and in the polymerase and other proteins that are associated with increased virulence and transmissibility in mammals [2–4].

In ferret experiments, H7N9 virus replicates well in the upper respiratory tract following intranasal inoculation, causes relatively mild illness, and is efficiently transmitted by direct contact, but less so by respiratory droplets [2, 3, 5]. Intratracheal inoculation of ferrets results in severe pneumonia and high mortality [6]. In a ferret model, therefore, H7N9 virus possesses a constellation of features that are intermediate between highly pathogenic H5N1 viruses and fully adapted but less virulent human influenza A viruses such as influenza A subtypes H3N2 and pandemic H1N1/2009 (pH1N1).

Despite meeting the criteria for a low pathogenic phenotype in birds, H7N9 virus has caused severe and fatal disease in humans [7]. However, the demographic profile of patients with H7N9 virus infection is unusual, with a high median age and an excess of males [8]. Although this might be due to age and sex differences in exposures to infected poultry or settings contaminated by infected poultry, the pattern differs markedly from H5N1 cases, and would also be consistent with age-dependent biological cofactors contributing to pathogenesis and disease severity [8]. An assessment of the clinical severity of human infections with H7N9 virus has concluded that many mild cases may have occurred and the overall symptomatic case fatality risk is estimated to be <3% [7]. Understanding the determinants of the severity of disease due to H7N9 virus infection is important both for the identification and clinical management of high-risk cases and for the purposes of public health risk assessment and contingency planning.

To assess whether the H7N9 virus genotype translates into a distinct clinical phenotype in humans, and to provide insights into the pathogenesis of H7N9 virus infection, we compared the risk factors, clinical presentation, and progression of patients hospitalized with H7N9, H5N1, and pH1N1 virus infections.

## METHODS

### Subject Ascertainment

All subjects with influenza virus infection reported in this manuscript were hospitalized patients. The patients with laboratory-confirmed H7N9 infection were all hospitalized in China between 25 February and 4 May 2013. The Chinese H5N1 cases represent all hospitalized cases of laboratory-confirmed H5N1 virus infection detected between 30 November 2003 and 8 February 2012. The Vietnamese H5N1 cases represent all hospitalized cases of laboratory-confirmed H5N1 virus infection detected between 25 December 2003 and 14 March 2009 [9]. A comparison of the Chinese and Vietnamese H5N1 cases showed similar demographic characteristics, underlying medical conditions, and behavioral risk factors (Supplementary Data). Patients with pH1N1 virus infection in China were ascertained through hospitals designated for the treatment of severe cases. The case definitions and time periods for ascertaining patients hospitalized with influenza A H5N1, H7N9, and pH1N1 virus infections are available in the Supplementary Data.

Clinical and laboratory data were abstracted retrospectively from original medical records for cases of H7N9, H5N1, and pH1N1 virus infections. Laboratory values were presented as medians with interquartile ranges and were dichotomized into normal or abnormal based on normal ranges for children and adults (Supplementary Table 1). Because the only subjects aged <29 days were 5 subjects with pH1N1 virus infection, and normal laboratory values are different in neonates compared with other age groups, we excluded all subjects aged <29 days from the assessment of laboratory results. We excluded pH1N1 cases from the analysis of signs and symptoms on admission as the ascertainment process for these cases required the presence of 1 or more symptoms, many of which were severe.

### Ethics Statement

The Chinese National Health and Family Planning Commission determined that the collection of data from H5N1, H7N9, and pH1N1 cases was part of public health investigations of emerging influenza outbreaks and was exempt from institutional review board assessment. The Science and Ethics Committee of the Ministry of Science and Technology of Vietnam approved the collection of clinical data from Vietnamese subjects with H5N1 virus infection.

### Risk Factors for Hospitalization and Death

To assess the importance of putative risk factors for hospitalization with each influenza A subtype, we estimated the relative risk of being hospitalized in subjects with and without risk factors. Data on the prevalence of each risk factor in the general Chinese population were used as denominators for the risk estimates and to weight (adjust) the overall relative risk estimates by age and sex. Data on age- and sex-specific population prevalence were available for coronary heart disease, chronic renal disease, diabetes, hypertension, smoking, and obesity; age-specific but not sex-specific population prevalence was available for asthma and chronic obstructive pulmonary disease (COPD) [10–13]. The definitions for these conditions are shown in the Supplementary Data. The age- and sex-stratified population prevalence of chronic heart disease (CHD; excluding isolated hypertension) was estimated from a study that recorded a prior history of hospitalization with coronary artery disease (A history of hospitalization for myocardial infarction or a surgical history of coronary balloon angioplasty, or coronary stent implantation or coronary artery bypass.) [10]. We assumed that the age distribution of coronary artery disease is a valid proxy for the age distribution of CHD. Where surveys assessed disease prevalence only in older adults, we assumed that prevalence was zero in those younger than the lower age limit of the survey. Because we were not able to source relevant baseline data for Vietnam, we have assumed that the age distribution of chronic diseases is similar in the Chinese and Vietnamese populations.

### Statistical Methods

We compared the characteristic of patients infected by different subtypes using Fisher exact test or χ^2^ test for comparing proportions and Wilcoxon signed-rank test for comparing medians of continuous variables. To evaluate the association between risk factors and the risk of hospitalization, Poisson regression was used to estimate the incidence rate ratios associated with each risk factor, adjusted for age and sex. The association between risk factors and the risk of death among hospitalized cases was assessed using multivariable logistic regression to estimate the odds ratios associated with each risk factor, adjusted for age and sex. In both analyses a spline function was used for age to allow for the possibly nonlinear effect of age on risk.

We used the Kaplan-Meier method to estimate survival curves for death and the hospitalized fatality risk. We used the same approach to estimate the time to invasive mechanical ventilation. The censoring time of each recovered or nonventilated patient was set to 90 days. The 95% confidence intervals (CIs) for the cumulative proportion of subjects requiring invasive ventilation and with a fatal outcome were estimated using bootstrapping with 1000 resamples.

We used maximum likelihood to estimate the distribution of the number of days of hospitalization, and compared alternative parametric distributions including γ, Weibull, and log-normal distributions, selecting the best parametric distribution on the basis of the Akaike information criterion.

## RESULTS

As of 6 August 2013, 133 laboratory-confirmed influenza A(H7N9) cases have been officially recorded in mainland China. Among these, 123 requiring hospitalization for medical reasons were included in this study [7]. Ten laboratory-confirmed mild cases were excluded [14]. Data were included for 119 patients hospitalized with H5N1 (Vietnam = 76; China = 43), and 3486 patients hospitalized with pH1N1.

The median age of subjects hospitalized with H7N9 was 63 years, compared to 26 years for H5N1 patients and 25 years for pH1N1 patients (*P* < .001). A higher proportion of H7N9 subjects were male compared with H5N1 (*P* = .019) or pH1N1 subjects (*P* = .001). Subjects hospitalized with H7N9 had the highest prevalence of chronic medical conditions traditionally associated with an increased risk of severe seasonal influenza disease (Table 1). CHD and diabetes were the commonest medical risk factors reported among H7N9 patients, and the prevalence of smoking and hypertension was higher in subjects with H7N9 compared with the other patient groups. Pregnancy was more common in subjects hospitalized with pH1N1.

**Table 1.** Characteristics of Subjects Hospitalized With Influenza A Virus Subtypes H7N9, H5N1, and pH1N1

Characteristic	H7N9^a^	H5N1	*P* Value	pH1N1	*P* Value
Age, y, median (range)	63 (4–91)	26 (1–75)	<.001	25 (0–100)	<.001
Interval from onset, admission days (IQR)	4 (3–6)	5 (3–6)	.155	4 (3–6)	.244
Male sex	87/123 (71%)	67/119 (56%)	.019	1937/3486 (56%)	.001
Any coexisting chronic medical conditions	42/105 (40%)	11/104 (11%)	<.001	748/3485 (21%)	<.001
Chronic heart disease	12/105 (11%)	1/102 (1%)	.001	147/3457 (4%)	.003
Chronic lung disease	10/105 (10%)	6/100 (6%)	.344	305/3397 (9%)	.849
Chronic renal disease	1/105 (1%)	1/102 (1%)	.984	91/3450 (3%)	.221
Chronic liver disease	5/105 (5%)	1/101 (1%)	.092	27/3478 (1%)	.002
Chronic neurological disease	3/105 (3%)	0/39 (0%)	.166	55/3472 (2%)	.356
Diabetes	18/105 (17%)	1/100 (1%)	<.001	185/3470 (5%)	<.001
Asthma	0/105 (0%)	0/0	NA	102/3442 (3%)	.013
Immune compromise	2/105 (2%)	1/100 (1%)	.586	86/3433 (3%)	.685
Hypertension	51/105 (49%)	2/41 (5%)	<.001	366/3479 (11%)	<.001
Malignancy	6/105 (6%)	1/41 (2%)	.375	92/3468 (3%)	.096
Pregnancy	2/105 (2%)	5/106 (5%)	.246	400/3436 (12%)	<.001
Smoking history	26/105 (25%)	10/88 (11%)	.015	541/3431 (16%)	.02
Obesity (BMI ≥30)	3/45 (7%)	0/10 (0%)	.265	175/2018 (9%)	.623

Any coexisting chronic medical conditions are any of the following: asthma, diabetes, chronic respiratory disease, chronic heart disease, chronic renal disease, chronic hepatic (liver) disease, chronic neurological disease, immune compromise (see Supplementary Data for definitions).

Abbreviations: BMI, body mass index; IQR, interquartile range; pH1N1, 2009 pandemic H1N1 virus.

^a^ Reference group.

Compared with subjects without CHD, the presence of CHD was associated with an increased risk of hospitalization with H7N9 (relative risk [RR], 9.68; 95% CI, 5.24–17.9; Table 2). CHD was also a risk factor for hospitalization with pH1N1 (RR, 16.51; 95% CI, 13.68–19.91). Hypertension was not associated with an increased risk of hospitalization in any group, whereas a history of smoking was associated with a reduced risk of hospitalization. Chronic renal disease was associated with a reduced risk of hospitalization in H7N9 and pH1N1 patients. Once patients were hospitalized, the odds of death were not significantly increased in subjects with any of the risk factors examined (Table 3).

**Table 2.** Age- and Sex-Adjusted Risk Factors for Hospitalization

Risk Factor^a^	Source of Baseline Prevalence Data	H7N9	H5N1	pH1N1
		RR (95% CI)^b^	RR (95% CI)^b^	RR (95% CI)^b^
Asthma^c^	[12, 15]	NC	NC	1.76 (1.43–2.15)
COPD^c^ (assume zero prevalence aged <40 y)	[11]	0.73 (.35–1.52)	4.25 (1.34–13.48)	1.76 (1.43–2.18)
Diabetes (assume zero prevalence aged <20 y)	[10]	1.11 (.67–1.87)	0.23 (.03–1.67)	1.11 (.94–1.30)
Chronic heart disease (assume zero prevalence aged <20 y)	[10]	9.68 (5.24–17.9)	NC	16.51 (13.68–19.91)
Chronic renal disease (assume zero prevalence aged <18 y)	[13]	0.07 (.01–.54)	NC	0.47 (.37–.58)
Hypertension (assume zero prevalence aged <20 y)	[10]	1.28 (.85–1.91)	0.45 (.10–1.99)	0.63 (.55–.71)
Smoking^d^	[10]	0.38 (.24–.60)	0.41 (.20–.88)	0.74 (.66–.84)
Obesity (BMI ≥30)^c^	[10]	1.16 (.36–3.74)	NC	2.42 (2.03–2.88)

Abbreviations: BMI, body mass index; CI, confidence interval; COPD, chronic obstructive pulmonary disease; NC, not calculable due to insufficient data; RR, relative risk.

^a^ See the Supplementary Data for definitions.

^b^ Adjusted for cubic spline for age (continuous) and sex where data were available.

^c^ Sex-specific data not available.

^d^ Restricted to subjects aged ≥20 years only.

**Table 3.** Age- and Sex-Adjusted Risk Factors for Death Among Hospitalized Patients

Risk Factor^a^	H7N9	H5N1	pH1N1
	Death^b^, OR (95% CI)	Death^b^, OR (95% CI)	Death^b^,OR (95% CI)
Asthma	NC	NC	0.24 (.06–1.01)
COPD	2.55 (.38–17.20)	0.92 (.12–6.83)	0.98 (.51–1.89)
Diabetes	3.68 (.97–14.03)	NC	0.85 (.51–1.44)
Chronic heart disease	0.96 (.18–5.17)	NC	1.22 (.72–2.08)
Chronic renal disease	NC	NC	1.56 (.86–2.80)
Hypertension	1.06 (.36–3.13)	0.24 (.01–6.92)	0.87 (.58–1.29)
Smoking	0.66 (.20–2.17)	1.23 (.25–5.99)	1.12 (.79–1.60)
Obesity (BMI ≥30)	NC	NC	0.96 (.59–1.56)

Abbreviations: BMI, body mass index; CI, confidence interval; COPD, chronic obstructive pulmonary disease; NC, not calculable due to insufficient data; OR, odds ratio.

^a^ See Supplementary Data for definitions.

^b^ Adjusted for cubic spline for age (continuous) and sex.

Signs and symptoms at hospital admission were compared for H7N9 and H5N1 cases. Subjects with H7N9 virus infection were more likely to report a fever, a productive cough, and hemoptysis than those with H5N1 virus infection (Table 4). Gastrointestinal symptoms were most common in H5N1 cases.

**Table 4.** Signs and Symptoms on Admission^a^

Sign or Symptom	H7N9	H5N1	*P* Value
Fever (temp ≥37.8)	99/105 (94%)	75/102 (74%)	<.001
Any cough	96/105 (91%)	89/106 (84%)	.097
Productive cough	59/104 (57%)	35/94 (37%)	.006
Dry cough	17/105 (16%)	45/94 (48%)	<.001
Yellow sputum	33/105 (31%)	10/61 (16%)	.029
Hemoptysis	25/105 (24%)	5/61 (8%)	.008
Myalgia	21/105 (20%)	12/50 (24%)	.572
Fatigue	38/105 (36%)	9/37 (24%)	.179
Shortness of breath	62/105 (59%)	54/93 (58%)	.889
Gastrointestinal symptoms	15/105 (14%)	17/53 (32%)	.01
Diarrhea	10/105 (10%)	6/50 (12%)	.64
Vomiting	4/105 (4%)	10/54 (19%)	.003
Nausea	6/105 (6%)	7/50 (14%)	.093
Central nervous system symptoms	4/105 (4%)	8/113 (7%)	.285

^a^ Or earliest available time point after admission.

The values of hematological, liver, and renal function tests, and markers of inflammation on admission are shown in Table 5. H7N9 and H5N1 patients showed similar patterns of elevated alanine aminotransferase, creatinine kinase, C-reactive protein, and lactate dehydrogenase, which were all significantly higher than in pH1N1 patients. Leukopenia and thrombocytopenia were equally common in patients with H7N9 and H5N1 virus infections, and more common than in those with pH1N1 virus infection. Lymphopenia was more common in patients with H7N9 compared with H5N1 (88% vs 55%; *P* < .001), and neutropenia was more common in H5N1 patients. Neutrophilia was equally common in H5N1 and pH1N1 patients, and least common in H7N9 patients.

**Table 5.** Laboratory Results on Admission^a^

Result	H7N9^b^	H5N1	*P* Value	pH1N1	*P* Value
White cell count	4.5 (2.9–6.2)	3.9 (2.5–7.1)	.805	6 (4.2–8.8)	<.001
Lymphocyte count	0.5 (0.3–0.7)	0.9 (0.6–1.4)	<.001	1 (0.6–1.5)	<.001
Neutrophil count	3.3 (2.2–5.4)	3 (1.5–5.4)	.203	4.3 (2.6–6.9)	.004
Platelet count	114 (82–147.5)	126 (86–196)	.203	173 (132–229.8)	.004
AST	53 (38–96.5)	100 (47–233)	.076	40 (26.4–68.5)	<.001
ALT	35.5 (24–64.5)	48.5 (29.5–99.5)	<.001	24 (15.6–44)	<.001
Serum creatinine	70.7 (58.3–85)	83 (54–100)	.028	62 (45.4–81)	<.001
CK	195 (96–562)	552 (126.5–939.8)	.255	120 (62–304)	<.001
CRP	65 (25–113)	51 (14.2–118.3)	.191	25.4 (7.9–75.5)	<.001
LDH	498 (388–661)	1025 (334.8–1832.5)	.525	307 (217–491)	<.001
Leukopenia	48/105 (46%)	54/107 (50%)	.489	736/3305 (22%)	<.001
Lymphopenia	88/99 (89%)	54/98 (55%)	<.001	1601/2891 (55%)	<.001
Neutropenia	13/103 (13%)	24/97 (25%)	.027	221/2891 (8%)	.086
Neutrophilia	5/103 (5%)	15/97 (15%)	.011	477/2891 (16%)	<.001
Thrombocytopenia	80/104 (77%)	69/105 (66%)	.073	1106/3066 (36%)	<.001
Elevated AST	54/103 (52%)	41/54 (76%)	.004	1165/3197 (36%)	.001
Elevated ALT	34/100 (34%)	25/52 (48%)	.093	668/3167 (21%)	.003
Elevated serum creatinine	11/103 (11%)	9/62 (15%)	.469	201/3054 (7%)	.129
Elevated CK	48/98 (49%)	13/20 (65%)	.188	1018/2951 (34%)	.004
Elevated CRP	83/92 (90%)	9/12 (75%)	.162	1193/1708 (70%)	<.001
Elevated LDH	89/98 (91%)	17/21 (81%)	.218	1617/2922 (55%)	<.001

Data are presented as median (IQR) or No. (%).

Abbreviations: ALT, alanine aminotransferase; AST, aspartate aminotransferase; CK, creatine kinase; CRP, C-reactive protein; IQR, interquartile range; LDH, lactate dehydrogenase; pH1N1, 2009 pandemic H1N1 virus.

^a^ Or earliest available time point after admission.

^b^ Reference group.

**Figure 1.**
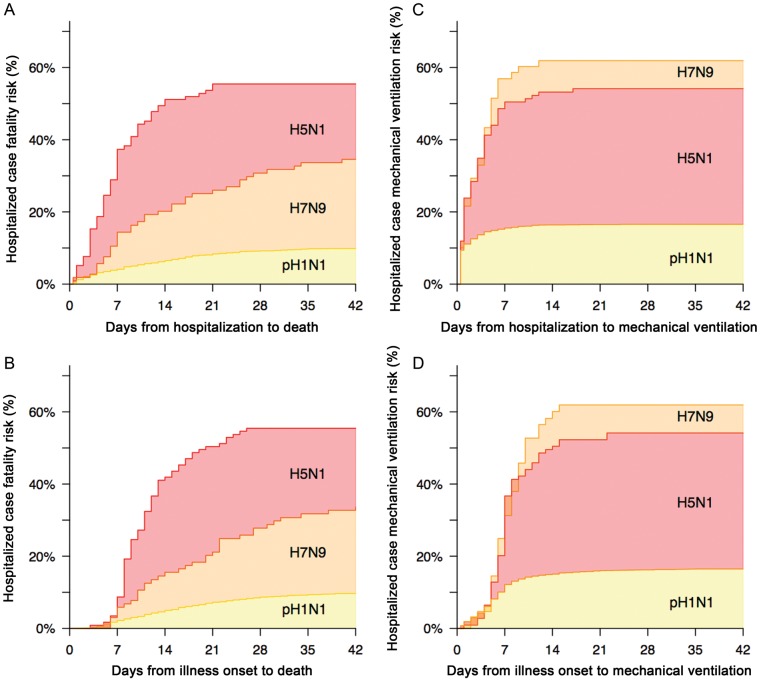
Case fatality risk and invasive ventilation risk in hospitalized patients. *A* and *B*, Case fatality risk by influenza A virus subtype and day of hospitalization (*A*) and day of illness onset (*B*). *C* and *D*, Invasive ventilation risk by influenza A virus subtype and day of hospitalization (*C*) and day of illness onset (*D*). Abbreviation: pH1N1, 2009 pandemic H1N1 virus.

**Figure 2.**
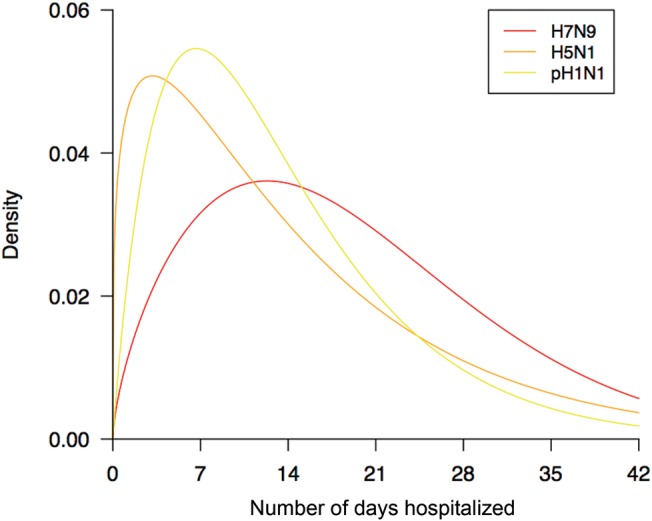
Distribution of the number of days of hospitalization for patients with H7N9, H5N1, and pH1N1.

The risk of invasive ventilation and death among hospitalized cases by influenza A virus subtype are shown in Figure 1. The cumulative proportion of hospitalized subjects requiring invasive ventilation differs between subtypes, reaching 62% (95% CI, 53%–71%) for H7N9, 54% (95% CI, 45%–63%) for H5N1, and 17% (95% CI, 15%–18%) for pH1N1. Among those ventilated, the interval from onset to invasive ventilation was a median of 7 days for both H7N9 and H5N1 cases (*P* = .651), and 6 days for pH1N1 cases. The hospitalized case fatality risk was highest for H5N1 (55%; 95% CI, 47%–64%) and death occurred earlier, with a median time from onset to death of 11 days for H5N1, compared with 15 days for pH1N1 patients (*P* = .154) and 18 days for H7N9 (*P* = .002). H7N9 patients were hospitalized for a longer duration than either H5N1 (*P* < .001) or pH1N1 patients (*P* < .001) (Figure 2).

## DISCUSSION

One of the most striking differences in this and other comparative analysis is the high median age of H7N9 patients [16]. This age distribution is unlikely to be due to differences in humoral immunity as the prevalence of neutralizing antibodies to H7N9 virus is probably low in all ages [17–20]. It might arise either because elderly people are more often exposed to the animal or environmental reservoir of H7N9 viruses, or because elderly people have a greater propensity to become infected or severely ill following exposure. After adjusting for the age- and sex-specific prevalence of chronic illnesses in the general Chinese population, we found that CHD was associated with an increased risk of hospitalization with H7N9 virus infection (RR, 9.68; 95% CI, 5.24–17.9). The age distribution of H7N9 patients may therefore be partially explained by an increased propensity in persons with CHD (who are mostly older) to develop severe disease following infection with H7N9 virus. The overrepresentation of males among H7N9 patients may also be partially explained by this association, because in China coronary heart disease is commoner in males than females (male prevalence, 0.74%; female prevalence, 0.51%) [10]. In agreement with our results, an age- and sex-matched case control study of 25 H7N9 cases has reported that the presence of a preexisting chronic medical condition (excluding hypertension) was associated with H7N9 disease (odds ratio, 5.1; 95% CI, 1.5–16.9) [21]. Although only 11% of H7N9 patients reported a history of CHD, unrecognized CHD may have been present in some individuals, and other unmeasured age-related factors, such as impaired innate and cell-mediated immunity, might also contribute to the observed age distribution of hospitalized H7N9 cases [17, 22, 23]. H7N9 viruses isolated from humans exhibit a mixed receptor specificity, binding both α2–6- and α2–3-linked sialidases [3, 4, 20]. H7N9 virus can infect cells of both the upper and lower respiratory tract of humans and ferrets, and disease in ferrets is more severe following intratracheal inoculation [5, 6, 20]. This raises the possibility that susceptibility of humans to severe H7N9 disease may be a consequence of an impaired ability to control virus replication in the lower respiratory tract.

A history of chronic renal disease was associated with a reduced risk of hospitalization with H7N9 virus infection, but the number of patients with this condition was small, so this finding should be interpreted with caution. A history of smoking was associated with a reduced risk of hospitalization with H7N9, H5N1, and pH1N1 virus infections. This is an unexpected finding that might be biased by inconsistent definitions and methods of ascertaining smoking history, which were not standardized in the clinical datasets.

The clinical presentation and laboratory indices at hospital admission are similar for H7N9 and H5N1 patients, except that a productive cough, hemoptysis, lymphopenia, and neutropenia were more common in H7N9 patients. Neutropenia, thrombocytopenia, and elevated liver enzymes are common in H5N1 patients and have been associated with more severe outcomes [9, 24–29]. A low absolute lymphocyte count has been associated with poor outcomes in patients hospitalized with pH1N1, H5N1, and severe acute respiratory syndrome [9, 30–32]. The hematological and serum chemistry abnormalities suggest that subjects hospitalized with H7N9 have a severe systemic illness. It remains to be determined if this is a consequence of severe pneumonia and poor tissue oxygenation or is the result of an excessive inflammatory response (as is seen with H5N1 virus infection) [33]. High levels of chemokines and cytokines have been identified in patients with H7N9 virus infection [20]. Extrapulmonary virus replication is an alternative explanation for the severity of hospitalized H7N9 cases, but H7N9 virus does not posses the polybasic amino acid motif at the HA cleavage site normally associated with extrapulmonary virus replication, and experimental H7N9 virus infection of ferrets has provided little evidence of systemic replication [2, 34, 35]. H7N9 viral RNA has been detected in the serum, urine, and feces of H7N9 patients but it is not known if this represents viral replication occurring outside of the respiratory tract [35].

Hospitalized H7N9 patients had a case fatality risk that was intermediate between pH1N1 and H5N1 patients, and a more protracted clinical course than either H5N1 or pH1N1 patients, with the longest median time to death and the longest hospitalization. Whether this reflects the natural history of severe H7N9 virus infection, patient characteristics, or differences in the clinical management of patients with severe H7N9 compared with H5N1 patients, including increased frequency of rescue modalities such as extracorporeal membrane oxygenation, is unknown.

The comparisons we have made are limited by a lack of standardization of the methods of case ascertainment and inclusion, and of the recording of clinical and other data. As such, the patients and data included in this study may be subject to unmeasured selection and information biases and differences in practices over time and between locations. However, we have tried to minimize these potential biases by restricting our analysis only to hospitalized subjects and to variables where data were available for a reasonable proportion of all cases. Although the H5N1 patients from China and Vietnam had very similar demographic characteristics, underlying medical conditions, and behavioral risk factors, there were some differences in clinical presentation (Supplementary Data), and we cannot exclude that the clinical phenotype of H5N1 virus infections may be heterogeneous. We used univariate analysis that adjusted for age and sex to explore possible risk factors for hospitalization with H7N9; interactions effects were not assessed and the estimated odds ratios and RRs might be confounded by other unmeasured confounders; as such, these risk factors should not be considered to be causal without further validation.

In conclusion, this comparative analysis shows that patients hospitalized with H7N9 virus infection share some risk factors with those hospitalized with pH1N1 infection but have a clinical profile more closely resembling that of H5N1 patients. The identification in H7N9 patients of known risk factors for severe seasonal influenza and the more protracted clinical course compared with H5N1 patients suggests that host factors may be an important contributor to the severity of H7N9 virus infection. This is consistent with the observation that there have probably been a large number of undetected mild H7N9 virus infections, and to date the patients with detected mild infection have been predominantly young (mean age, 13 years) [7, 14]. H7N9 virus has recently reemerged in China. People with chronic medical conditions that are traditionally associated with a higher risk of severe complications following seasonal influenza virus infection should be targeted for preventive interventions and for early treatment with antiviral drugs should they develop a respiratory illness.

## Supplementary Data

Supplementary materials are available at *Clinical Infectious Diseases* online (http://cid.oxfordjournals.org). Supplementary materials consist of data provided by the author that are published to benefit the reader. The posted materials are not copyedited. The contents of all supplementary data are the sole responsibility of the authors. Questions or messages regarding errors should be addressed to the author.

Supplementary Data
