# Supplementary material for: Comparison of Patients Hospitalized With Influenza A Subtypes H7N9, H5N1, and 2009 Pandemic H1N1
Source: Clin Infect Dis. 2014 Jan 31;58(8):1095–103. doi: 10.1093/cid/ciu053 (PMC3967826; doi:10.1093/cid/ciu053)
Supplement: Supplementary Data [file supp_58_8_1095__index.html]

Comparison of patients hospitalized with influenza A H7N9, H5N1, and 2009 pandemic H1N1 — Comparison of Patients Hospitalized With Influenza A Subtypes H7N9, H5N1, and 2009 Pandemic H1N1 — Comparison of Patients Hospitalized With Influenza A Subtypes H7N9, H5N1, and 2009 Pandemic H1N1 — Supplementary Data 

# Comparison of Patients Hospitalized With Influenza A Subtypes H7N9, H5N1, and 2009 Pandemic H1N1

## Supplementary Data

Supplementary Data

**Files in this Data Supplement:**

- Supplementary Data - Docx file
