# Supplementary material for: Comparison of Patients Hospitalized With Influenza A Subtypes H7N9, H5N1, and 2009 Pandemic H1N1
Source: Clin Infect Dis. 2014 Jan 31;58(8):1095–103. doi: 10.1093/cid/ciu053 (PMC3967826; doi:10.1093/cid/ciu053)
Supplement: Supplementary Data [file supp_ciu053_ciu053supp.docx]

**Supplementary materials**

**Case definitions for ascertainment of patients hospitalized with influenza A virus infection.**

***Patients hospitalized with H7N9 virus infection***

A confirmed H7N9 case was defined as a hospitalized patient with respiratory specimens that tested positive for H7N9 virus by any of the following: isolation of H7N9 virus or positive results by real-time RT-PCR assay for H7N9; or a four-fold or greater rise in H7N9 virus antibody titer based on testing of an acute serum specimen (collected 7 days or less after symptom onset) and a convalescent serum specimen collected at least two weeks later.[^1^](#_ENREF_1) The patients with H7N9 virus infection were all hospitalized between February 25^th^ and May 4th 2013.

***Patients hospitalized with H5N1 virus infection***

For the China H5N1 data set, a confirmed case of H5N1 virus infection was defined as a hospitalized patient with pneumonia or influenza-like illness (ILI) and laboratory evidence of H5N1 virus infection diagnosed by viral isolation or reverse transcription polymerase chain reaction (RT-PCR) testing of respiratory specimens, or a four-fold or greater increase in H5N1 virus antibody titer in paired acute and convalescent sera.[^2^](#_ENREF_2) For the Vietnam H5N1 data set, a confirmed case of H5N1 virus infection was defined as a hospitalized patient with laboratory evidence of H5N1 virus infection diagnosed by viral isolation or RT-PCR testing of respiratory specimens.[^3^](#_ENREF_3) The Chinese patients with H5N1 virus infection were all hospitalized between November 30 2003 and February 8 2012. The Vietnamese patients with H5N1 virus infection were all hospitalized between December 25 2003 and March 14 2009. Tables S1-3 compare subjects hospitalized with H5N1 virus infection in China and Vietnam

**Table S1. Characteristics of subjects hospitalized with H5N1 virus infection in China and Vietnam**

|  | H5N1 China (ref group) | H5N1 Vietnam | P-value | H5N1 combined |
| --- | --- | --- | --- | --- |
| Median age (range) | 26 (2, 62) | 25 (1, 75) | 0.668 | 26 (1, 75) |
| Interval from onset – admission days (IQR) | 5 (4,6.5) | 4 (2,6) | 0.880 | 5 (3,6) |
| Male gender | 22/43 (51%) | 45/76 (59%) | 0.396 | 67/119 (56%) |
| Any Co-existing chronic medical conditions | 3/41 (7%) | 8/63 (13%) | 0.373 | 11/104 (11%) |
| Chronic heart disease | 1/41 (2%) | 0/61 (0%) | 0.175 | 1/102 (1%) |
| Chronic lung disease | 1/41 (2%) | 5/59 (8%) | 0.186 | 6/100 (6%) |
| Chronic renal disease | 1/41 (2%) | 0/61 (0%) | 0.175 | 1/102 (1%) |
| Chronic liver disease | 0/41 (0%) | 1/60 (2%) | 0.306 | 1/101 (1%) |
| Chronic neurological disease | 0/39 (0%) | NA | NA | 0/39 (0%) |
| Diabetes | 0/41 (0%) | 1/59 (2%) | 0.303 | 1/100 (1%) |
| Asthma | NA | NA | NA | NA |
| Immune compromise | 0/41 (0%) | 1/59 (2%) | 0.303 | 1/100 (1%) |
| Hypertension | 2/41 (5%) | NA | NA | 2/41 (5%) |
| Malignancy | 1/41 (2%) | NA | NA | 1/41 (2%) |
| Pregnancy | 4/41 (10%) | 1/65 (2%) | 0.053 | 5/106 (5%) |
| Smoking history | 8/41 (20%) | 2/47 (4%) | 0.021 | 10/88 (11%) |
| Obesity (BMI ≥30) | 0/6 (0%) | 0/4 (0%) | 1.000 | 0/10 (0%) |

Any Co-existing chronic medical conditions is any of the following: Asthma, Diabetes, Chronic respiratory disease, Chronic heart disease, Chronic renal disease, Chronic hepatic (liver) disease, Chronic neurological disease, Immune compromise (see Supplementary materials for definitions).

**Table S2. Signs and symptoms on admission* of subjects hospitalized with H5N1 virus infection in China and Vietnam**

| Sign or symptom | H5N1 China (ref group) | H5N1 Vietnam | P-value | H5N1 combined |
| --- | --- | --- | --- | --- |
| Fever (temp >= 37.8) | 33/37 (89%) | 42/66 (64%) | 0.003 | 75/102 (74%) |
| Any cough | 28/37 (76%) | 61/69 (88%) | 0.096 | 89/106 (84%) |
| Productive cough | 15/37 (41%) | 20/57 (35%) | 0.594 | 35/94 (37%) |
| Dry cough | 16/37 (43%) | 29/57 (51%) | 0.469 | 45/94 (48%) |
| Yellow sputum | 8/18 (44%) | 2/43 (5%) | <0.001 | 10/61 (16%) |
| Hemoptysis | 2/18 (11%) | 3/43 (7%) | 0.601 | 5/61 (8%) |
| Myalgia | 8/37 (22%) | 4/13 (31%) | 0.514 | 12/50 (24%) |
| Fatigue | 9/37 (24%) | NA | NA | 9/37 (24%) |
| Shortness of breath | 12/37 (32%) | 42/56 (75%) | <0.001 | 54/93 (58%) |
| Gastrointestinal symptoms | 7/37 (19%) | 10/16 (62%) | 0.002 | 17/53 (32%) |
| Diarrhea | 1/37 (3%) | 5/13 (38%) | 0.001 | 6/50 (12%) |
| Vomiting | 3/37 (8%) | 7/17 (41%) | 0.005 | 10/54 (19%) |
| Nausea | 3/37 (8%) | 4/13 (31%) | 0.057 | 7/50 (14%) |
| Central nervous system symptoms | 2/37 (5%) | 6/76 (8%) | 0.621 | 8/113 (7%) |

* Or earliest available time point after admission. NA = Not available

**Table S3. Laboratory results on admission* of subjects hospitalized with H5N1 virus infection in China and Vietnam**

| Median (inter-quartile range) | H5N1 China (ref group) | H5N1 Vietnam | P-value | H5N1 combined |
| --- | --- | --- | --- | --- |
| White cell count | 3.6 (2.2,5.5) | 4.1 (2.5,9.2) | 0.126 | 3.9 (2.5,7.1) |
| Lymphocyte count | 0.9 (0.5,1.1) | 1 (0.6,1.5) | 0.106 | 0.9 (0.6,1.4) |
| Neutrophil count | 2.9 (1.5,4.4) | 3.1 (1.5,7.7) | 0.184 | 3 (1.5,5.4) |
| Platelet count | 101 (86,135) | 134 (95.2,210.5) | 0.010 | 126 (86,196) |
| Aspartate aminotransferase | 79 (58,150) | 175 (47,426) | 0.085 | 100 (47,233) |
| Alanine aminotransferase | 28.4 (21.8,46.2) | 74.5 (40.8,143.5) | <0.001 | 48.5 (29.5,99.5) |
| Serum creatinine | 82.6 (59.9,99.2) | 80.5 (54,102.5) | 0.999 | 83 (54,100) |
| CK | 537 (114,927.5) | 664 (187,872) | 0.824 | 552 (126.5,939.8) |
| CRP | 174 (99.7,175.5) | 24 (11.5,51) | 0.103 | 51 (14.2,118.3) |
| LDH | 711 (328,1148) | 1967 (1101.2,2768.8) | 0.025 | 1025 (334.8,1832.5) |
| n/N (%) |  |  |  |  |
| Leukopenia | 21/37 (57%) | 33/70 (47%) | 0.344 | 54/107 (50%) |
| Lymphopenia | 19/30 (63%) | 35/68 (51%) | 0.274 | 54/98 (55%) |
| Neutropenia | 7/29 (24%) | 17/68 (25%) | 0.928 | 24/97 (25%) |
| Neutrophilia | 0/29 (0%) | 15/68 (22%) | 0.001 | 15/97 (15%) |
| Thrombocytopenia | 30/35 (86%) | 39/70 (56%) | 0.001 | 69/105 (66%) |
| Elevated aspartate aminotransferase | 17/21 (81%) | 24/33 (73%) | 0.486 | 41/54 (76%) |
| Elevated alanine aminotransferase | 4/20 (20%) | 21/32 (66%) | 0.001 | 25/52 (48%) |
| Elevated serum creatinine | 2/24 (8%) | 7/38 (18%) | 0.256 | 9/62 (15%) |
| Elevated CK | 7/11 (64%) | 6/9 (67%) | 0.888 | 13/20 (65%) |
| Elevated CRP | 4/5 (80%) | 5/7 (71%) | 0.733 | 9/12 (75%) |
| Elevated LDH | 10/13 (77%) | 7/8 (88%) | 0.539 | 17/21 (81%) |

* Or earliest available time point after admission. NA = Not available

***Patients hospitalized with pandemic H1N1/2009 virus infection***

The case definition was an individual hospitalized between September 1 and December 31 2009 with pH1N1 virus infection confirmed by testing respiratory specimens with real-time RT-PCR assay and any one of the following features: persistent high fever ＞3 days; severe cough, [purulent](app:ds:purulent) [sputum](app:ds:sputum), bloody sputum, or chest pain; [tachypnea](app:ds:tachypnea), shortness of breath, or cyanosis; a disturbance of consciousness; severe vomiting or diarrhea leading to [dehydration](app:ds:dehydration); pneumonia on chest radiography; increased CK, CK-MB.[^4^](#_ENREF_4) The patients with pH1N1 virus infection were all hospitalized between between May 12 and December 31, 2009

**Definitions used for chronic diseases and other risk factors.**

Table S1 shows the source and definitions of prevalence of chronic diseases and risk factors that were used to estimate the relative risk of hospitalisation.

**Table S4. Source and definitions of prevalence of chronic diseases and risk factors**

| **Risk factor** | **Source of baseline**  **prevalence data** | **Definition** |
| --- | --- | --- |
| Asthma (children) | [^5^](#_ENREF_5) | Self reported answer to question “Has your child ever had asthma”. |
| Asthma (adults) | [^6^](#_ENREF_6) | Self-reported |
| COPD | [^7^](#_ENREF_7) | Post-bronchodilator FEV1/ FVC of less than 70% |
| Diabetes | [^8^](#_ENREF_8) | Fasting plasma glucose ≥ 7.0mmol/l |
| Chronic heart disease | [^8^](#_ENREF_8) | A history of hospitalization for myocardial infarction or a surgical history of coronary balloon angioplasty, or coronary stent implantation or coronary artery bypass. |
| Chronic renal disease | [^9^](#_ENREF_9) | Estimated glomerular filtration rate less than 60 mL/min per 1·73 m2 or the presence of albuminuria |
| Hypertension | [^8^](#_ENREF_8) | Average blood pressure between two measurements ≥140/90 mmHg, or a previous diagnosis of hypertension |
| Smoking | [^8^](#_ENREF_8) | Smoking one or more cigarettes daily for at least 1 year. |
| Obesity | [^8^](#_ENREF_8) | BMI ≥30kg/m^2^ |

**Definitions used to define the presence of chronic disease in patients hospitalized with influenza A.**

***Chronic respiratory disease***

Chronic obstructive pulmonary disease (COPD) including chronic bronchitis and emphysema; bronchiectasis, cystic fibrosis, interstitial lung fibrosis, pneumoconiosis and bronchopulmonary dysplasia. Excludes asthma.

- ***Chronic heart disease***
- Congenital heart disease, cardiomyopathy as the result of prolonged hypertension (hypertension alone in the absence of associated heart disease was not considered a risk factor), chronic heart failure, individuals requiring regular medication and/or follow-up for ischaemic heart disease.
- ***Chronic renal disease***
- Chronic renal failure,  nephrotic syndrome, renal transplantation

***Chronic hepatic (liver) disease***

Cirrhosis, biliary atresia, chronic hepatitis

***Chronic neurological disease***

Stroke, neuromuscular diseases that leads to impaired respiratory function or aspiration risk such as cerebral palsy or myasthenia gravis**.**

- ***Immune compromise***
- Immunodeficiencies related to use of immunosuppressive drugs (e.g. chemotherapy) or systemic steroids; Asplenia or splenic dysfunction (sickle cell anemia); Human Immunodeficiency Virus infection and Acquired Immune Deficiency Syndrome.

**Age specific normal ranges for laboratory tests.**

Table S2 shows the age specifc reference ranges used to define abnormalities in blood results. Neonates (subejcts aged <29 days) were excluded from this analysis.

**Table S5. Age specific reference ranges used to define abnormalities in blood results.**

| **Measure** | **Units** | **Cut-off** |
| --- | --- | --- |
| Leukopenia | 10^9^ cells per liter | 2 months – 2 years: <5  >2 years: <4 |
| Lymphopenia | 10^9^ cells per liter | 2-11 months: <4.0  1-11 years: <1.5  12+ years: <1 |
| Neutropenia | 10^9^ cells per liter | All ages: <1.5 |
| Neutrophilia | 10^9^ cells per liter | All ages: >8.5 |
| Thrombocytopenia | 10^9^ cells per liter | All ages: <150 |
| Elevated aspartate aminotransferase | U per liter | All ages: >50 |
| Elevated alanine aminotransferase | U per liter | All ages: >50 |
| Elevated serum creatinine | μmol per liter | All ages: >120 |
| Elevated CK | U per liter | All ages: >200 |
| Elevated CRP | mg/l | All ages: >10 |
| Elevated LDH | U per liter | <7 years: >400  7-15 years: >300  16+ years: >250 |

Source: Mayo Medical Laboratories, pediatric test reference values.

<http://www.mayomedicallaboratories.com/test-info/pediatric/refvalues/reference.php>

Accessed 07 June 2013

**References**

1. Li Q, Zhou L, Zhou M, et al. Preliminary Report: Epidemiology of the Avian Influenza A (H7N9) Outbreak in China. The New England journal of medicine 2013.

2. Yu H, Gao Z, Feng Z, et al. Clinical characteristics of 26 human cases of highly pathogenic avian influenza A (H5N1) virus infection in China. PloS one 2008;3:e2985.

3. Liem NT, Tung CV, Hien ND, et al. Clinical features of human influenza A (H5N1) infection in Vietnam: 2004-2006. Clinical infectious diseases : an official publication of the Infectious Diseases Society of America 2009;48:1639-46.

4. Yu H, Feng Z, Uyeki TM, et al. Risk factors for severe illness with 2009 pandemic influenza A (H1N1) virus infection in China. Clinical infectious diseases : an official publication of the Infectious Diseases Society of America 2011;52:457-65.

5. Zhao J, Bai J, Shen K, et al. Self-reported prevalence of childhood allergic diseases in three cities of China: a multicenter study. BMC public health 2010;10:551.

6. National Center for Chronic and Noncommunicable Disease Control and Prevention. Report on chronic disease risk factor surveillance in China. Beijing; 2007.

7. Zhong N, Wang C, Yao W, et al. Prevalence of chronic obstructive pulmonary disease in China: a large, population-based survey. American journal of respiratory and critical care medicine 2007;176:753-60.

8. Yang ZJ, Liu J, Ge JP, Chen L, Zhao ZG, Yang WY. Prevalence of cardiovascular disease risk factor in the Chinese population: the 2007-2008 China National Diabetes and Metabolic Disorders Study. European heart journal 2012;33:213-20.

9. Zhang L, Wang F, Wang L, et al. Prevalence of chronic kidney disease in China: a cross-sectional survey. Lancet 2012;379:815-22.
